# Supplementary material for: Anti-Apoptotic c-FLIP Reduces the Anti-Tumour Activity of Chimeric Antigen Receptor T Cells
Source: Cancers (Basel). 2022 Oct 4;14(19):4854. doi: 10.3390/cancers14194854 (PMC9564159; doi:10.3390/cancers14194854)
Supplement: Supplementary file 1 [file cancers-14-04854-s001.zip › cancers-1907268-supplementary.pdf]

cancers

Supplementary Materials: Anti-apoptotic c-FLIP reduces the anti-tumour activity of chimeric antigen receptor T cells

Grace Min Yi Tan, Aarati Poudel, Ali Hosseini Rad S.M. and Alexander Donald McLellan

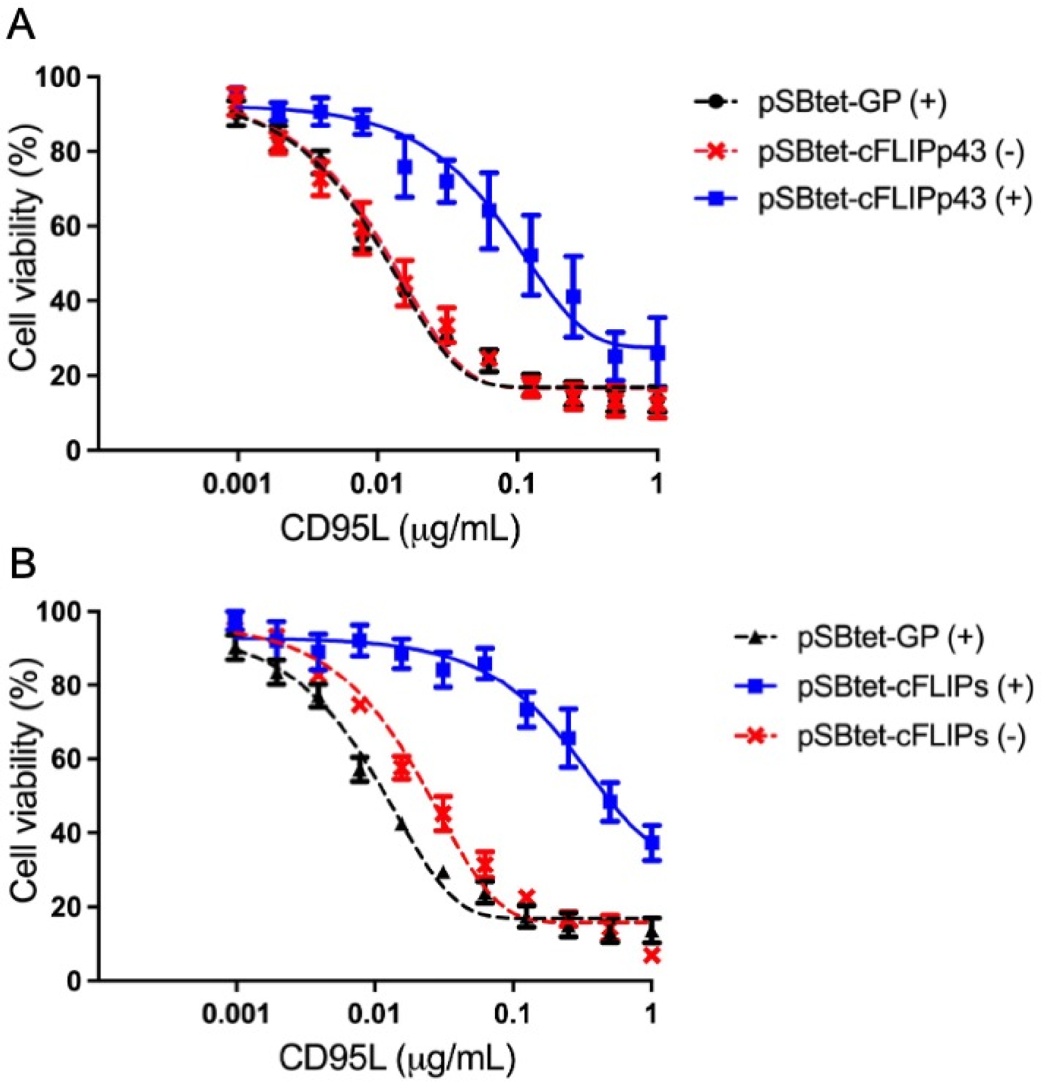

Figure S1. Tet-On induction of c-FLIP protects Jurkat T cells against cell-death induced by CD95L challenge. Jurkat cells were transposed with Tet-On pSB-Tet-On [1,2] constructs using the SB100X transposase vector containing no insert (pSB-GP) or the same vector with the indicated insert. Stable cell lines were generated using puromycin selection. Stably transfected Jurkat cells expressing c-FLIP isoforms with (+) or without (-) induction with 5 μg/mL doxycycline treatment were challenged with 500 ng/mL of LZ-CD95L for 24h to mimic AICD. Cell viability was determined by resazurin assay [3]. Cells were incubated with resazurin solution and quantified as previously described [3]. IC_{50} curve were plotted as relative cell viability percentage against untreated cells. Error bars represent mean ± SEM of triplicate samples (n=3).

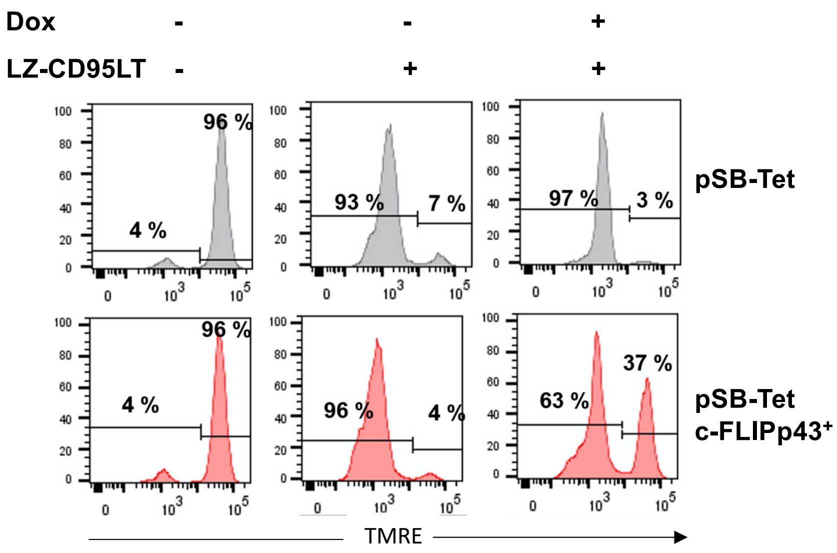

Figure S2. Tet-On induction of c-FLIP protects Jurkat T cells against mitochondrial depolarisation mediated by CD95L challenge. Jurkat cells were transfected with Tet-On pSB-Tet-On [1,2] constructs and selected using puromycin. Stably transfected Jurkat cells expressing c-FLIP with (+) or without (-) induction with 5 µg/mL doxycycline treatment were challenged with 500 ng/mL of LZ-CD95L for 24h to mimic AICD. Cell viability was determined by TMRE assay [3]. Cells were stained with TMRE as previously described [4,5]. Histograms displays cell count of GFP^+ gated cells TMRE staining (x-axis). $TMRE^+$ events (right gate) represent cells with high Ψ_M while $TMRE^-$ (left gate) are cells with depolarised mitochondria. Flow charts are a representative of three independent repeats.

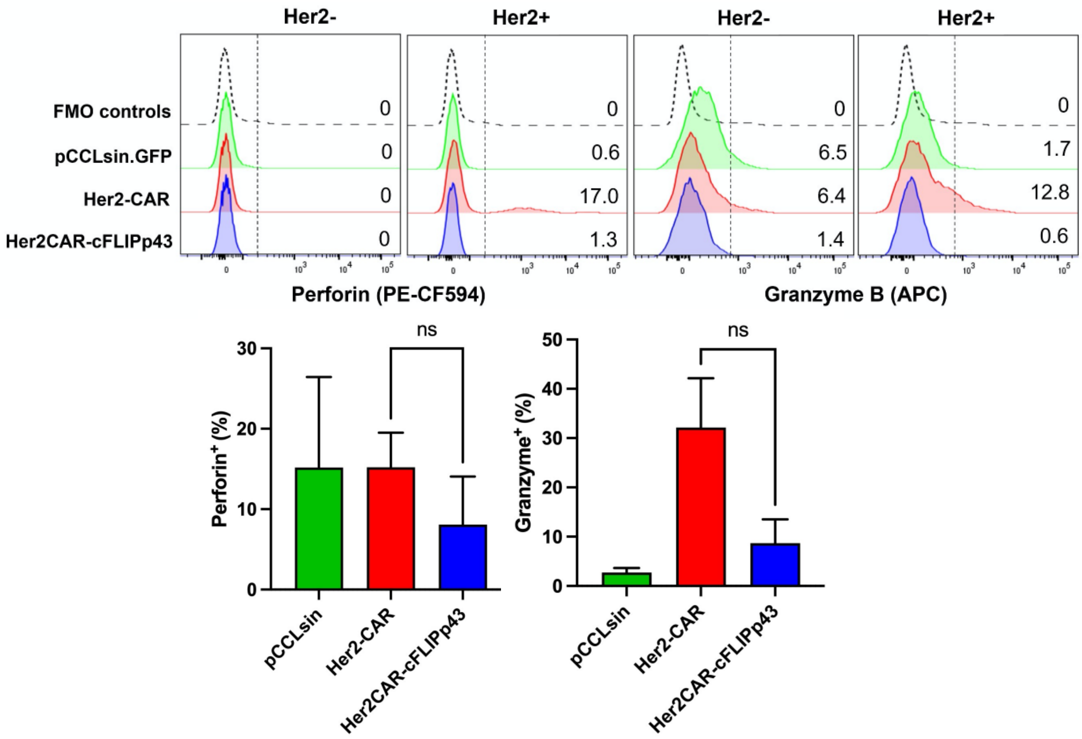

Figure S3. Perforin and granzyme B production of c-FLIP-expressing CAR T cells. Negative control, pCCLsin, CAR control Her2-CAR or Her2CAR-cFLIPp43 transduced primary human T cells were cocultured at a 2:1 ratio for six hours with Her2-target MCF-7 with four final hours of Brefeldin A treatment. The cells were fixed, and intracellularly stained with perforin and granzyme B antibody for flow cytometric analysis. Representative donor flow plots (n=3) of cell count versus perforin or granzyme B with Her2+ antigen and without Her2+ antigen. (lower). Bar graph values represent the mean values ± SEM from three independent experiments where each experiment indicates different donor (n=3). Statistical analysis: two-tailed paired t test.

References

1. Kowarz, E.; Loscher, D.; Marschalek, R. Optimized Sleeping Beauty transposons rapidly generate stable transgenic cell lines. *Biotechnol. J.* **2015**, *10*, 647–653, doi:10.1002/biot.201400821.
2. Ali Hosseini Rad, S.M.; Poudel, A.; Tan, G.M.Y.; McLellan, A.D. Optimisation of Tet-On inducible systems for Sleeping Beauty-based chimeric antigen receptor (CAR) applications. *Sci. Rep.* **2020**, *10*, 13125, doi:10.1038/s41598-020-70022-0.
3. Bouwer, A.L.; Saunderson, S.C.; Caldwell, F.J.; Damani, T.T.; Pelham, S.J.; Dunn, A.C.; Jack, R.W.; Stoitzner, P.; McLellan, A.D. NK cells are required for dendritic cell-based immunotherapy at the time of tumor challenge. *J. Immunol.* **2014**, *192*, 2514–2521.
4. McLellan, A.D.; Terbeck, G.; Mengling, T.; Starling, G.C.; Kiener, P.A.; Gold, R.; Brocker, E.B.; Leverkus, M.; Kampgen, E. Differential susceptibility to CD95 (Apo-1/Fas) and MHC class II-induced apoptosis during murine dendritic cell development. *Cell Death Differ.* **2000**, *7*, 933–938, doi:10.1038/sj.cdd.4400734.
5. McLellan, A.; Heldmann, M.; Terbeck, G.; Weih, F.; Linden, C.; Brocker, E.B.; Leverkus, M.; Kampgen, E. MHC class II and CD40 play opposing roles in dendritic cell survival. *Eur. J. Immunol.* **2000**, *30*, 2612–2619, doi:10.1002/1521-4141(200009)30:9<2612::AID-IMMU2612>3.0.CO;2-G.
